# The Functional DPP4 Receptor Is an Indispensable Factor Mediating the Immune Performance of Mucosal Vaccines for Middle East Respiratory Syndrome

**DOI:** 10.1155/tbed/2303502

**Published:** 2025-06-10

**Authors:** Zhenshan Wang, Xiaojun Hu, Shen Wang, Hongyu Sun, Yongkun Zhao, Na Feng, Tiecheng Wang, Guixue Hu, Jianzhong Wang, Xianzhu Xia, Feihu Yan

**Affiliations:** ^1^College of Veterinary Medicine, Jilin Agricultural University, Changchun 130022, China; ^2^Changchun Veterinary Research Institute, State Key Laboratory of Pathogen and Biosecurity, Key Laboratory of Jilin Province for Zoonosis Prevention and Control, Chinese Academy of Agricultural Sciences, Changchun 130022, China; ^3^Jiangsu Co-Innovation Center for Prevention and Control of Important Animal Infectious Diseases and Zoonoses, Yangzhou University, Yangzhou 225009, China

**Keywords:** animal model, binding affinity, dipeptidyl peptidase-4, Middle East respiratory syndrome, mucosal vaccines

## Abstract

Mucosal vaccines are powerful tools for combatting emerging infectious diseases, particularly mucosal-associated pathogens. However, one of the main bottlenecks in developing mucosal vaccines is the lack of accurate animal models. In this study, a vesicular stomatitis virus (VSV)-vectored Middle East respiratory syndrome coronavirus (MERS-CoV) mucosal vaccine was designed for investigations. Compared with the VSV backbone, rVSVΔG-MERS-S exhibited altered cellular tropism, as determined by MERS-S. In wild-type (WT) C57BL-6J mice and hamsters, the nasal spray of rVSVΔG-MERS-S was poorly immunogenic. In contrast, rVSVΔG-MERS-S was highly immunogenic in transgenic mice (hDPP4 mice) and hDPP4-transduced hamsters harboring the functional MERS-CoV receptor. Compared with those of WT C57BL-6J mice, the nasal spray of rVSVΔG-MERS-S resulted in effective antigen-presenting cell (APC) priming, Tfh-GcB-plasma cell (pC) proliferation, and robust humoral and cellular responses, together with the activation of antiviral signaling pathways in hDPP4 mice. Similarly, rVSVΔG-MERS-S was highly immunogenic in alpacas and rhesus monkeys, which are naturally susceptible to MERS-CoV and harbor the effective DPP4 receptor. The alignment of hDPP4 receptors in these animals revealed that L294, I295, and R336 in DPP4 are key residues contributing to differences in sensitivity across species. Consistently, a high binding affinity was observed between human, alpacas, and rhesus monkey DPP4 receptors and MERS-CoV receptor binding domains (RBDs) compared with that of mice and hamster. Overall, this proof-of-concept study not only guides the selection of appropriate animal models for the evaluation of mucosal vaccines of MERS but also provides evidence that functional receptors DPP4 in animal models are prerequisites for the immune performance of the MERS mucosal vaccine.

## 1. Introduction

Emerging and re-emerging infectious diseases are highly detrimental to public health and have a considerable impact on the global economy. Typically, severe acute respiratory syndrome coronavirus (SARS-CoV), severe acute respiratory syndrome coronavirus 2 (SARS-CoV-2), and Middle East respiratory syndrome coronavirus (MERS-CoV) are highly transmissible beta-coronaviruses (Beta-CoVs) and are associated with waves of outbreak [1–3]. To combat these highly transmissible respiratory pathogens, a main consideration is the development of mucosal vaccines, which enable the establishment of both local mucosal and systemic immune responses [4, 5]. Mucosal vaccines are potential candidates for blocking the transmission of mucosal-associated pathogens [6]. However, a main process in developing mucosal vaccines is the selection of an appropriate animal model. Previous studies have shown species-associated sensitivity differences in animal models when evaluating the efficacy of mucosal vaccines [7–10]. Although differences in functional receptors have been speculated to be responsible for this phenomenon, further investigations are needed to uncover this point of view in detail.

To this end, a recombinant vesicular stomatitis virus (VSV) (rVSV) was prepared, namely, rVSVΔG-MERS-S. According to previous research, rVSVΔG-MERS-S is a replication-competent virus that expresses solely a foreign glycoprotein, and the tropism of rVSVΔG-MERS-S was determined via MERS-S [11, 12]. Consequently, rVSVΔG-MERS-S could be a promising mucosal vaccine candidate since it recapitulates the natural infection process of MERS-CoV, followed by triggering inflammation and the immune response through the natural production of pathogen-associated molecular patterns (PAMPs). The subsequent problem lies in how to choose the correct animal model to provide the most authentic safety and efficacy data for the target species.

As expected, rVSVΔG-MERS-S was confirmed to be a promising mucosal vaccine candidate for MERS-CoV in this study. More importantly, the function of the hDPP4 receptor in triggering the immune response to mucosal vaccines was further revealed. Specifically, these include receptor-associated pathogen recognition, immune response initiation, and progression. Moreover, key residues and binding affinities that may contribute to the differences in sensitivity across species were resolved. These details could guide the rational selection of animal models for the evaluation of mucosal vaccines in future studies.

## 2. Methods and Materials

### 2.1. Cells and Animals

BHK-21 (ATCC, CCL-10), Vero E6 (ATCC, CRL-1586), and HuH-7 (ATCC, JCRB-0403) cells were cultured at 37°C in Dulbecco's Modified Eagle Medium (DMEM) (GIBCO, Grand Island, NY, USA) containing 10% fetal bovine serum (Thermo Fisher, MA, USA) and penicillin (100 U/mL)–streptomycin (100 mg/mL) (Thermo Fisher, MA, USA) with 5% CO_2_ for virus propagation and titration. C57BL/6J mice (*Mus musculus*, female, 6–8 weeks of age, SPF) were purchased from Beijing Vital River Laboratory Animal Technology Co., Ltd. hDPP4 mice (cat. no. NM-HU-190042-*Mus musculus*, female, 6–8 weeks of age, SPF) were purchased from Shanghai Model Organisms Center, Inc. Rhesus macaques and alpacas were provided by the Wildlife Rescue and Breeding Centre of Jilin Province.

### 2.2. Animal Ethics Statement

All animal procedures were reviewed and approved by the Animal Experiment Committee of Changchun Veterinary Research Institute, China (Assurance Number: IACUC-DWZX-2022-030).

### 2.3. rVSVΔG-MERS-S Generation and Amplification

rVSVΔG-MERS-S is a replication-competent virus in which the glycoprotein gene of VSV G was replaced with that of MERS-S (GenBank accession no. KF186567.1). Enhanced green fluorescent protein (eGFP) was added to an additional transcription unit between the G and L genes in the VSV genome. Recombinant plasmid construction and recombinant virus rescue were carried out as previously described [7, 13]. Briefly, rVSVs were rescued via transfection with rVSV plasmids and supporting plasmids encoding N, P, L, and G of VSV via the calcium phosphate (Thermo Fisher, MA, USA) method in BHK-21. Sixty hours posttransfection, the rVSVs in the supernatants were collected. Serial passages were performed in Vero E6 cells. rVSVΔG-MERS-S was collected and stored at −80°C.

### 2.4. Indirect Immunofluorescence

Vero E6 cells at 80% confluence were infected with rVSVΔG-MERS-S at a multiplicity of infection (MOI) of 0.01. The cells were fixed with cold acetone 36 h after infection. Following inactivation, the appropriate MERS S protein-specific antibody (Sino Biological, Inc., CAT#40069-T62) was diluted in phosphate-buffered saline (PBS) for 1 h at room temperature. Then, the samples were incubated for another hour with an antispecies rabbit-specific antibody (goat antirabbit IgG [H + L] FITC, BAO7161595) in PBS for 30 min at room temperature. Eventually, the nuclei were stained with the appropriate diluted DAPI in PBS for 10 min at room temperature. The samples were analyzed via a Thermo Fisher Scientific microscope.

### 2.5. Western Blot

The rVSVΔG-MERS-S-infected cell lysates were separated via 10% SDS-PAGE and subsequently electrotransferred to nitrocellulose membranes. The nitrocellulose membrane was blocked for 2 h at 4°C with blocking buffer (Beyotime Biotechnology). The samples were subsequently incubated with an anti-S rabbit polyclonal antibody (Sino Biological, Inc.) (diluted 1:3000) overnight at 4°C. The target bands were detected with an HRP-conjugated antirabbit antibody (goat antirabbit IgG [H + L] HRP, BWD-BS13278) at a 1:5000 dilution. The samples were examined with a Tanon 5200 chemiluminescence imaging system.

### 2.6. Growth Kinetics

Vero E6 cells were infected with rVSVΔG-MERS-S at an MOI of 0.01. Supernatants were collected at the indicated time points (12, 24, 36, 48, 60, and 72) postinfection and titered by TCID_50_ via the Reed–Muench method.

### 2.7. Gene Expression Knockdown by RNA Interference

To knock down the expression of the human DPP4 in HuH-7 cells, Ambion Validated Silencer Selected siRNA (Thermo Fisher, Waltham, MA) was used to target the specific sequence of DPP4 or clathrin. Briefly, siRNA targeting DPP4 or irrelevant siRNA (negative control, Ambion cat no. 4390843) was prepared on 96-well cell carrier plates (Perkin Elmer, Waltham, MA). Next, 35 μL of OptiMEM (Invitrogen, Oregon, USA) containing 0.15 μL of Lipofectamine RNAiMAX transfection reagent (Invitrogen, Oregon, USA) was mixed with 60 μL of OptiMEM containing 1 × 10^4^ Vero E6 cells. The cell–RNAiMAX mixture was then added to the wells. The cells were incubated for 48 h to knock down gene expression, after which the cells were infected with VSVΔG-eGFP-MERS.

### 2.8. Animal Experiments

Animal grouping and experimental design were summarized in Table S1. For safety evaluations, suckling mice (7 days old), C57BL-6J mice (6 weeks old, female), golden hamsters (6 weeks old, female), and SD rat (6 weeks old, female) were intraperitoneally injected with rVSVΔG-MERS-S (106 TCID50/mL, 100 μL) (*n* = 5 per group). Survival rate and weight were carried for 14 days. For the mouse experiments, the animals were divided into four groups (*n* = 5 per group). The hDPP4 mouse vaccination group (10^6^ TCID_50_/mL, 100 μL, rVSVΔG-MERS-S), hDPP4 mouse control group (DMEM, 100 μL), C57BL-6J mouse vaccination group (10^6^ TCID_50_/mL, 100 μL, rVSVΔG-MERS-S), and C57BL-6J mouse control group (DMEM, 100 μL) were used. For the hamster experiment, the same immunization protocol was used for the mice (*n* = 5 per group). The hDPP4 hamsters were intranasally transduced with 5 × 10^8^ pfu of Ad5-hDPP4 5 days prior to vaccination. Then, hDPP4-transduced hamsters or wild-type (WT) hamsters were grouped and immunized as the similar protocol described in mice. All animals received a single dose of vaccination. At days 0, 7, and 21 postvaccination, blood was collected from each group for serum separation, and the samples were subjected to antibody measurement. For SIgA and cytokine detection, another four groups of mice were prepared as has been described (*n* = 15 per group, *n* = 5 per time point).

For the alpaca and rhesus monkey experiments, the same immunization protocol was adapted as that used for the mice (*n* = 3 per group). On days 7, 14, and 21 postvaccination, blood was collected from each group for serum separation, and the samples were subjected to antibody measurement.

### 2.9. Enzyme-Linked Immunosorbent Assay

The MERS-CoV S protein (1 µg/100 µL/well) was coated onto 96-well ELISA plates (Corning Costar, Corning, NY, USA) overnight at 4°C. The samples were washed three times with PBST and blocked for 2 h at 37°C with PBS containing 1% BSA. The immune sera or bronchoalveolar lavage fluid (BALF) were serially diluted in PBS containing 0.1% BSA and incubated for 1 h at 37°C with the immobilized spike antigen. Thereafter, the samples were washed three times with PBST, and the plates were incubated at 37°C for 1 h with the appropriate HRP-labeled goat antimouse or goat antimonkey IgG (or IgA) antibodies (goat antimouse IgG [H + L] HRP, BWD-BS12478, goat antimouse IgA alpha chain HRP, Abcam, ab97235, goat antimonkey IgG [H + L] HRP, TransGen, HS201-01). The ELISA plates were washed three times with PBST and then incubated for 10 min at room temperature with 100 µL/well of 3,3′5,5′-tetramethylbenzidine (TMB) peroxidase substrate. After color development was stopped with 50 µL/well H_2_SO_4_, the plate was read at 450 nm (Bio-Rad, Hercules, CA, USA).

### 2.10. Enzyme-Linked Immunospot Assay

An ELISPOT assay was performed to evaluate vaccine-induced T-cell responses in mice. Briefly, 7 days after immunization, according to the manufacturer's instructions, the splenocytes of the mice were cultured in DMEM supplemented with 5% FBS and 1% penicillin‒streptomycin and stimulated with 10 µg/mL receptor binding domain (RBD) protein for 48 h at 37°C and 5% CO_2_. Concanavalin (Thermo Scientific, USA) was used as a positive control. Images of the spots were acquired and counted with a Mabtech IRIS FluoroSpot/ELISpot reader, and the data were analyzed with GraphPad Prism.

### 2.11. Neutralization Assay

The serum neutralizing antibody levels were determined via rVSV-MERS-eGFP. All the serum was heat-inactivated at 56°C for 30 min, and then the serum was diluted in 96-well plates with twofold serial dilutions (from 1:20 to 1:20,480) and mixed with 100 TCID_50_ of VSV-MERS. The transfected cells were subsequently inoculated for 60 min at 37°C. After incubation, 50 µL of the preincubated mixture was added to Vero E6 cells.

### 2.12. Mesoscale Discovery

Mouse serum and alveolar lavage fluid were collected for testing. The mesoscale discovery assay (Univ#K15048D-X, China) was carried out according to the manufacturer's instructions. The plates were analyzed on a Sector Imager 2400 system, and cytokine concentrations were calculated on the basis of the standard curve generated in Discovery Workbench 4.0.12 software with a four-parameter logistic nonlinear regression analysis.

### 2.13. For Flow Cytometry Assays

Mice were divided into four groups (*n* = 5 per group): the hDPP4 mouse vaccination group (10^6^ TCID_50_/mL, 100 μL, rVSVΔG-MERS-S), the hDPP4 mouse control group (DMEM, 100 μL), the C57BL-6J mouse vaccination group (10^6^ TCID_50_/mL, 100 μL, rVSVΔG-MERS-S), and the C57BL-6J mouse control group (DMEM, 100 μL). For antigen-presenting cell (APC) detection, the lungs were harvested at 1 day postvaccination (dpv) (*n* = 5 per group). For Tfh-GcB-plasma cell (pC) detection, splenocytes were harvested at 7 dpv (*n* = 5 per group).

Briefly, mouse tissues were collected, physically separated, and ground in PBS (pH 7.4); the cells were resuspended in PBS containing 0.2% BSA, transferred to a centrifuge tube through a 40-μm nylon filter, centrifuged at 600 × *g*, and washed with PBS containing 0.2% (wt/vol) BSA. Red blood cells were removed via lysis buffer. After two washes with PBS, single-cell suspensions in PBS were counted. In total, 2 × 10^6^ cells were stained with flow cytometric antibodies (Table S2). After incubation for 30 min at 4°C, the cells were washed twice with PBS containing 0.2% BSA. Finally, the stained cells were analyzed via a BD Air-Fusion instrument. Flow cytometry data were analyzed via FlowJo V10 (BD, NY, USA).

### 2.14. DPP4 Alignment

DPP4 from mice, rhesus monkeys, humans, alpacas, and hamsters (accession numbers: NM_001159543.1, NM_001039190.1, NM_001935.3, XM_006196217.3, and NM_001310571.1) were aligned via MEGA 7.0.20. Differential sites at the receptor binding motif are marked.

### 2.15. Binding Kinetic Measurement by Biolayer Interferometry (BLI)

The streptavidin (SA) biosensor was immersed in a biotinylated RBD (10 µg/mL) solution for 300 s to obtain the baseline. The sensor was then immersed in kinetic buffer (2*⁣*^*∗*^PBST and 0.1% BSA) with nanobodies added over 120 s, which was diluted twice continuously from 200 to 12.5 nM. The dissociation of the nanobodies was detected in kinetic buffer for 180 s, and the affinity constant (KD) was determined via Data Analysis12.0.

### 2.16. Statistical Analyses

GraphPad Prism 8.0. software (GraphPad Software Inc., San Diego, CA, USA) was used to analyze the data. Kolmogorov–Smirnov test and/or Shapiro–Wilk test were applied to verify the normal distribution. Normally distributed data are expressed as the mean ± standard error of the mean (SEM). Significant differences between groups were determined using ANOVA. In cases that the assumption of normality cannot be verified, data are expressed as median ± interquartile range (IQR). Nonparametric Kruskal–Wallis test and multiple comparisons were performed. Survival curve-associated data were analyzed using log-rank analysis. *p* < 0.05 was considered to indicate statistical significance. Significance levels were defined as *⁣*^*∗*^*p* < 0.05, *⁣*^*∗∗*^*p* < 0.01, *⁣*^*∗∗∗*^*p* < 0.001, and *⁣*^*∗∗∗∗*^*p* < 0.0001.

## 3. Results

### 3.1. Identification and Characterization of rVSVΔG-MERS-S

**Figure 1:**
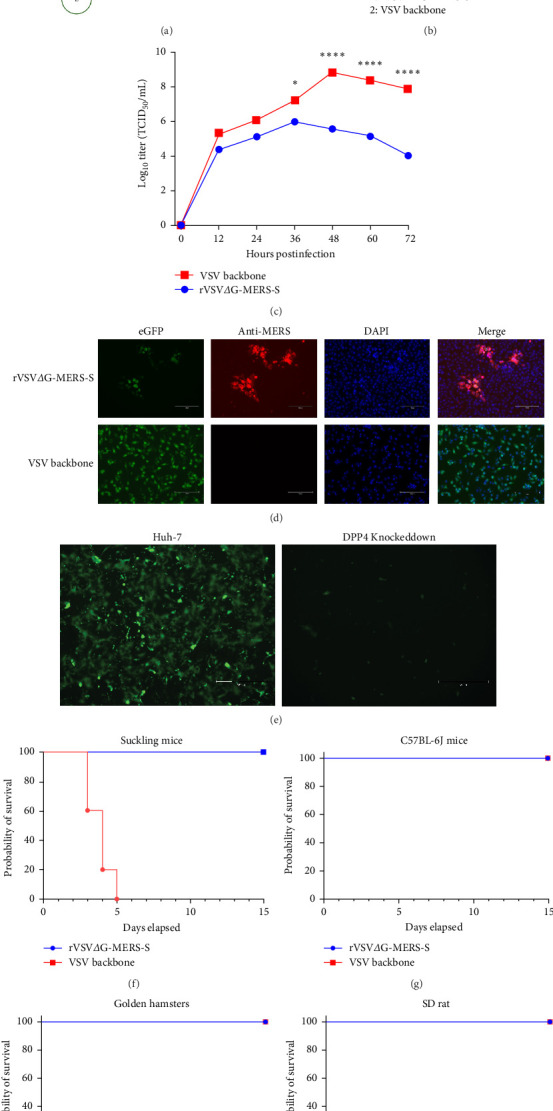
Characterization of rVSVΔG-MERS-S. (a) Schematic diagram depicting the genomic organization of PVSVΔG-MERS-S (shown in Figures 3–5) and the rescue process of rVSVΔG-MERS-S. (b) Western blot analysis of the rVSVΔG-MERS-S and VSV backbone control. (c) Growth kinetics of rVSVΔG-MERS-S and the VSV backbone. Vero E6 cells were infected with the rVSVΔG-MERS-S or VSV backbone at passage 5 at an MOI of 0.01. The TCID_50_/mL of each virus is indicated at each time point. (d) Indirect immunofluorescence detection of rVSVΔG-MERS-S via MERS-S-specific antibodies. (e) Proliferation of rVSVΔG-MERS-S in HuH-7 cell lines harboring the DPP4 receptor and DPP4 knockdown cells. (f) Fourteen days of successive observation of survival rate in sucking mice, of which VSV backbone causes 100% lethality while rVSVΔG-MERS-S was avirulent. (g–i) Fourteen days of successive observation of survival rate in C57BL-6J mice, golden hamsters, and SD rats (*n* = 5 per group). Significance levels were defined as *⁣*^*∗*^*p* < 0.05 and *⁣*^*∗∗∗∗*^*p* < 0.0001.

Using reverse genetic approaches, rVSVΔG-MERS-S was obtained by replacing the glycoprotein genes of the VSV backbones with those of MERS-S (Figure 1a). Replication-competent rVSVΔG-MERS-S was used as the mucosal immunogen for further studies. The recombinant virus rVSVΔG-MERS-S was identified by indirect immunofluorescence, Western blotting, and growth kinetics (Figure 1b–d). Western blotting and indirect immunofluorescence revealed that the recombinant viruses were recognized by a MERS glycoprotein-specific antibody, and a specific band at ~170 kDa or specific fluorescence signals was detected. In terms of growth kinetics, rVSVΔG-MERS-S presented the highest titer of 10^6^ TCID_50_/mL at 48 h postinfection (Figure 1c). In HuH-7 cell lines harboring the DPP4 receptor, rVSVΔG-MERS-S effectively proliferated, whereas when DPP4 was knocked down by siRNA, the propagation of rVSVΔG-MERS-S was significantly reduced (Figure 1e). With successive passages of rVSVΔG-MERS-S to the 20th generation, the supernatant of virion RNA was subjected to sequencing via MERS-S-specific primers, which revealed that no mutations had accumulated. In both sucking mice, C57BL-6J mice, golden hamsters, and SD rats, intraperitoneal injection of rVSVΔG-MERS-S did not lead to deaths or weight lose (Figure 1f–I, Figure S1). In contrast, VSV backbone led to 100% lethality in sucking mice (Figure 1f). Taken together, these results suggest that the recombinant virus rVSVΔG-MERS-S was rescued successfully and was applicable to be a mucosal vaccine candidate.

### 3.2. rVSVΔG-MERS-S Was Poorly Immunogenic in WT Mice and Hamsters But Induced Rapid, Robust MERS-Specific Humoral Immune Responses in hDPP4 Mice and hDPP4-Transduced Hamsters

**Figure 2:**
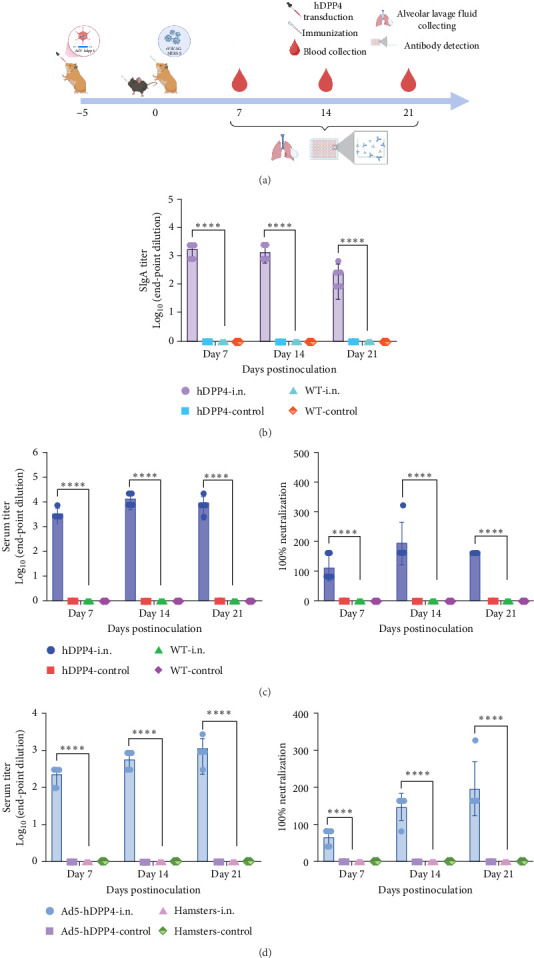
Humoral immune response triggered by rVSVΔG-MERS-S in hDPP4 mice and WT C57BL-6J mice. (a) Timeline and experimental schedule. In hDPP4 transduce hamsters, Ad5-hDPP4 was given at 5 days prior to immunization. At day 0, wild-type mice, hamsters, hDPP4 mice, and hDPP4-transduced hamsters were given a single dose of rVSVΔG-MERS-S via the IN spray route (10^6^ TCID_50_/mL, 100 μL, *n* = 5 per group). Blood was collected at 7, 14, and 21 dpi, and humoral immune responses were measured. Wild-type mice, hamsters, hDPP4 mice, and hDPP4-transduced hamsters vaccinated with the same volume of DMEM were used for the control group. (b) SIgA value detected at 7, 14, and 21 dpi in mice bronchoalveolar lavage fluid (*n* = 5 per group). (c) Binding antibodies and neutralizing antibodies in mice (*n* = 5 per group). (d) Binding antibodies and neutralizing antibodies in hamsters (*n* = 5 per group). Significance levels were defined as *⁣*^*∗∗∗∗*^*p* < 0.0001.

To investigate the immunogenicity of rVSVΔG-MERS-S, a single dose of rVSVΔG-MERS-S was administered via the nasal spray route to WT C57BL-6J mice, hamsters, hDPP4 mice, and hDPP4-transduced hamsters (Figure 2a). In the control group, the same volume of DMEM was added. BALF were collected at 7, 14, and 21 dpi for the detection of SigA. Blood samples were collected at 7, 14, and 21 dpi and subjected to measurement of MERS-CoV-specific binding and neutralizing antibodies. Compared to WT mice, rVSVΔG-MERS-S induced significantly increased MERS-CoV-specific SIgA in BALF (*p* < 0.0001), with endpoint titers greater than 10^3^ at 7 and 14 dpi and 10^2^–10^3^ at 21 dpi (Figure 2b). When rVSVΔG-MERS-S was given to WT mice and hamsters, neither the binding antibody nor the neutralizing antibody was converted during the 14-day follow-up observation (Figure 2c,d). In hDPP4 mouse hDPP4-transduced hamsters vaccinated with rVSVΔG-MERS-S, virus-neutralizing antibodies rapidly converted at 7 dpv and increased to a high reciprocal titer of 160−320 at 14 dpv (Figure 2c,d). Similarly, MERS-CoV-specific binding antibodies were elevated at 7 dpv and increased to a reciprocal titer of 21870 at 14 dpv (Figure 2c,d). Together, these results illustrated a distinct response to mucosal vaccination with rVSVΔG-MERS-S in WT animals and hDPP4 transgene/transduced animals.

### 3.3. rVSVΔG-MERS-S Promotes APC Priming and Tfh-GcB-pC Activation in hDPP4 Mice

**Figure 3:**
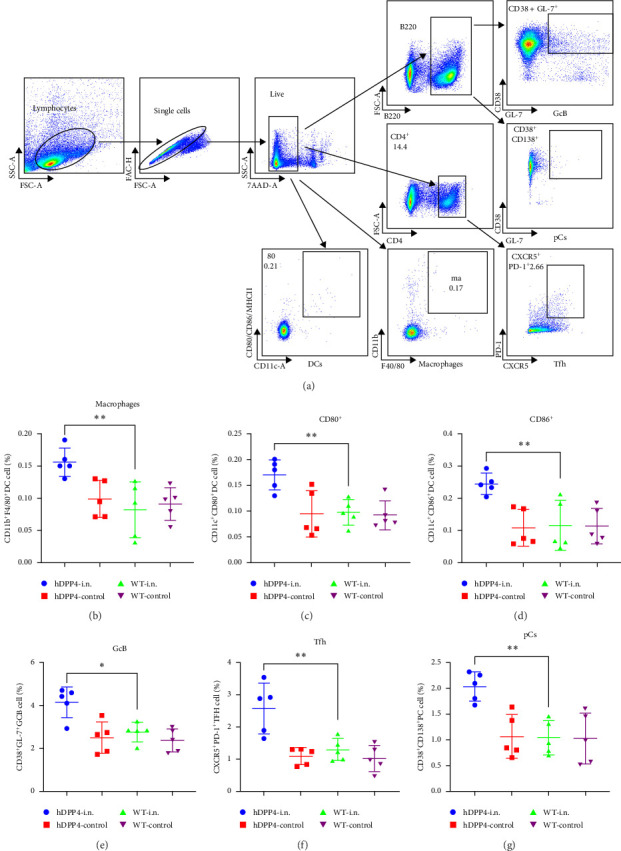
APC, Tfh, GcB, and pC activation induced by rVSVΔG-MERS-S. The animals were divided into four groups (*n* = 5 per group), namely, the hDPP4 vaccination group (10^6^ TCID_50_/mL, 100 μL, rVSVΔG-MERS-S), hDPP4 control group 1 (100 μL of DMEM), C57BL-6J vaccination group (10^6^ TCID_50_/mL, 100 μL, rVSVΔG-MERS-S), and C57BL-6J control group (100 μL of DMEM). Immune cells in the lungs or spleens of the animals were harvested at 1 or 7 days postvaccination (dpv) (*n* = 5 at each time point) and subjected to flow cytometry analysis. (a) Flow cytometry gating strategy. (b) Activation of CD11b+F40/80+ macrophages in the lungs. (c, d) Activation of CD11c^+^CD86^+^ and CD11c^+^CD80^+^ dendritic cells (DCs) in the lungs of animals at 1 dpv. (e) Activation of CD4^+^PD-1^+^CXCR5^+^ Tfh cells in the splenocytes of both groups at 7 dpv. (f) Activation of CD4^+^CD38^+^GL-7^+^ GcB cells in the splenocytes of both groups at 7 dpv. (g) Activation of CD45^+^CD38^+^CD138^+^ plasma cells (pCs) in the splenocytes of both groups at 7 dpv. The data are expressed as the mean ± SEM. Significance levels were defined as *⁣*^*∗*^*p* < 0.05, *⁣*^*∗∗*^*p* < 0.01, ⁣^*∗∗∗*^*p* < 0.001, and *⁣*^*∗∗∗∗*^*p* < 0.0001.

Since a differential response to the nasal spray of rVSVΔG-MERS-S was observed between WT mice and hDPP4 mice, mechanical insights into the rapid and robust humoral immune response were investigated via flow cytometry. Studies were carried out by analyzing APCs and antibody production associated with the Tfh-GcB-pC axis, and the results were compared between WT mice and hDPP4 mice. The splenocytes were labeled with the antibodies listed in Table S2 and gated according to the protocols described in Figure 3a.

Innate immune analyses were carried out in spleens. At 24 h postvaccination, a rapid increase in CD11c^+^CD86^+^, CD11c^+^CD40^+^, and CD11c^+^MHCII^+^ DC cells and CD11b^+^F40/80^+^ macrophages were noted in the nasal spray rVSVΔG-MERS-S-vaccinated hDPP4 mice compared with those in the WT and control groups (Figure 3b–d) (*p* < 0.01 in both the CD11c^+^CD80^+^ group, the CD11b^+^F40/80^+^ group, and the CD11c^+^CD86^+^ group) (Figure 3b–d). Consequently, compared with WT mice, rVSVΔG-MERS-S mice exhibited early and effective activation of APCs. At 7 dpv, the numbers of Tfh, GcB, and pCs in both groups of splenocytes were analyzed. As expected, significantly more CD4^+^PD-1^+^CXCR5^+^ Tfh cells, CD45^+^CD38^+^GL-7^+^ GcB, and CD45^+^CD38^+^CD138^+^ pCs were observed in the rVSVΔG-MERS-S-vaccinated hDPP4 mice than in the WT mice (*p* < 0.01) (Figure 3e–g). These results suggested the active proliferation of antibody production-associated APCs-Tfh-GcB-pC axis in rVSVΔG-MERS-S-vaccinated hDPP4 mice, while these immune cells showed no response in WT mice. Taken together, these results provide evidence that rVSVΔG-MERS-S triggers a rapid humoral immune response under the basis of functional DPP4 receptor by regulating the APC-Tfh-GcB-pC axes.

### 3.4. rVSVΔG-MERS-S Elicits a Cellular Immune Response in hDPP4 Mice

**Figure 4:**
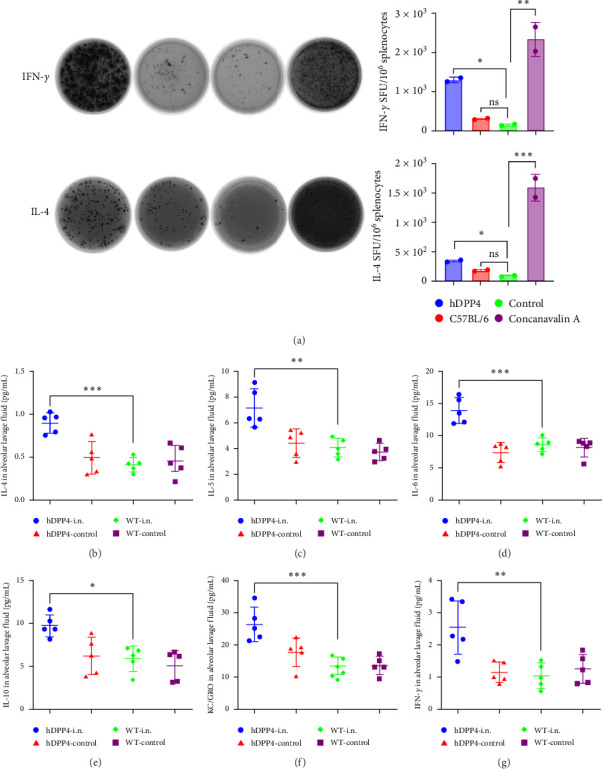
Cellular immune response elicited by rVSVΔG-MERS-S. (a) Representative cytokines (IL-4 and IFN-γ) in the splenocytes of both groups were detected via ELISpot. (b–g) Representative Th1 and Th2 cytokine levels in the lavage fluid of both groups were measured by MSD (*n* = 5 per group). Significance levels were defined as *⁣*^*∗*^*p* < 0.05, *⁣*^*∗∗*^*p* < 0.01, *⁣*^*∗∗∗*^*p* < 0.001, and *⁣*^*∗∗∗∗*^*p* < 0.0001.

Furthermore, representative cytokines were detected via ELISpot after MERS-CoV glycoprotein priming for 36 h. Compared with those in WT mice, MERS-CoV-specific IFN-γ and IL-4 were induced in hDPP4 mice (Figure 4a, *p* < 0.05). The representative cytokines in BALF were detected by MSD. Compared with those in WT mice, Th1 and Th2 cytokines, including IL-4, IL-5, IL-6, IL-10, KC-GRO, and IFN-γ, were significantly elevated in the hDPP4 group (*p*  < 0.001, *p* < 0.01, *p* < 0.001, *p* < 0.05, *p* < 0.001, and *p* < 0.01, respectively) (Figure 4b–g). These results suggest that rVSVΔG-MERS-S elicits cellular immune responses in hDPP4 mice at both the local and systemic levels.

### 3.5. Transcriptome Activation of Antiviral Pathways in the Lung After rVSVΔG-MERS-S Vaccination

**Figure 5:**
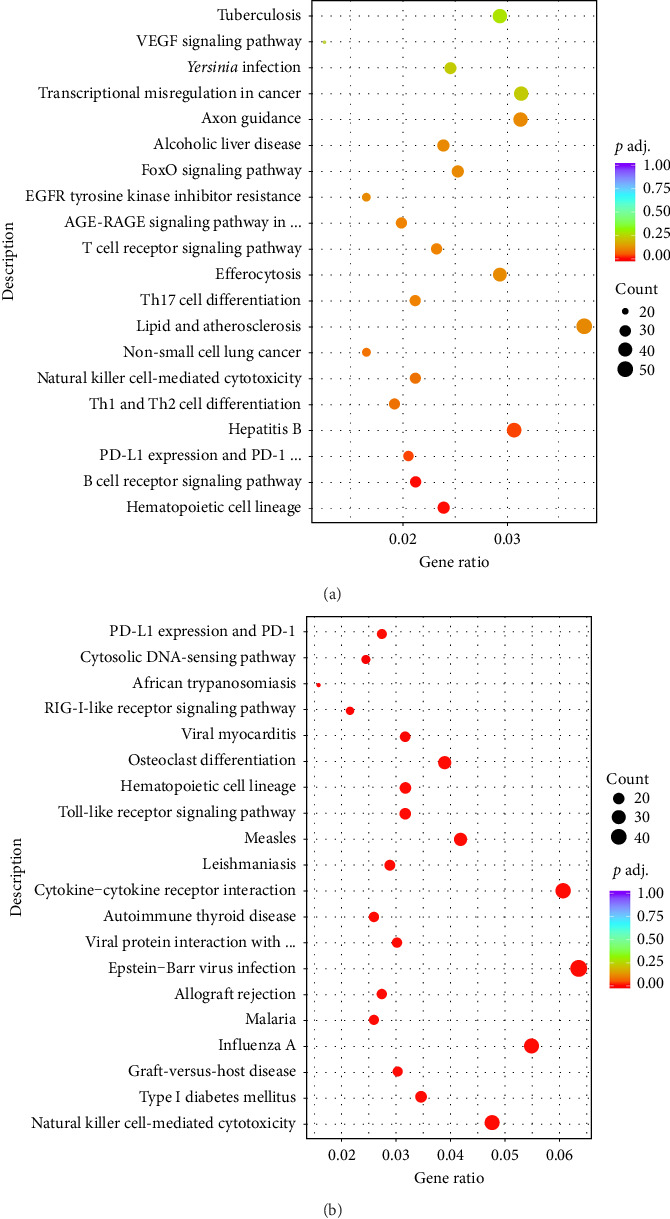
(a, b) Scatter plot of the results of the GO enrichment analysis of the top upregulated genes at 24 h after nasal spray vaccination with rVSVΔG-MERS-S in the lungs of hDPP4 and C57BL-6J mice (*n* = 5 per group).

To better understand the profile at the in situ level, transcriptome analysis was conducted on the lungs of rVSVΔG-MERS-S. According to transcriptome sequencing and GO and KEGG differential gene enrichment analyses at 24 hpi, the genes associated with the innate immune response, mucosal immune response, and humoral response that were most strongly upregulated were actively involved in the lungs of the hDPP4 vaccination group (Figure 5).

### 3.6. rVSVΔG-MERS-S Was Immunogenic in MERS-CoV-Susceptible Animals

**Figure 6:**
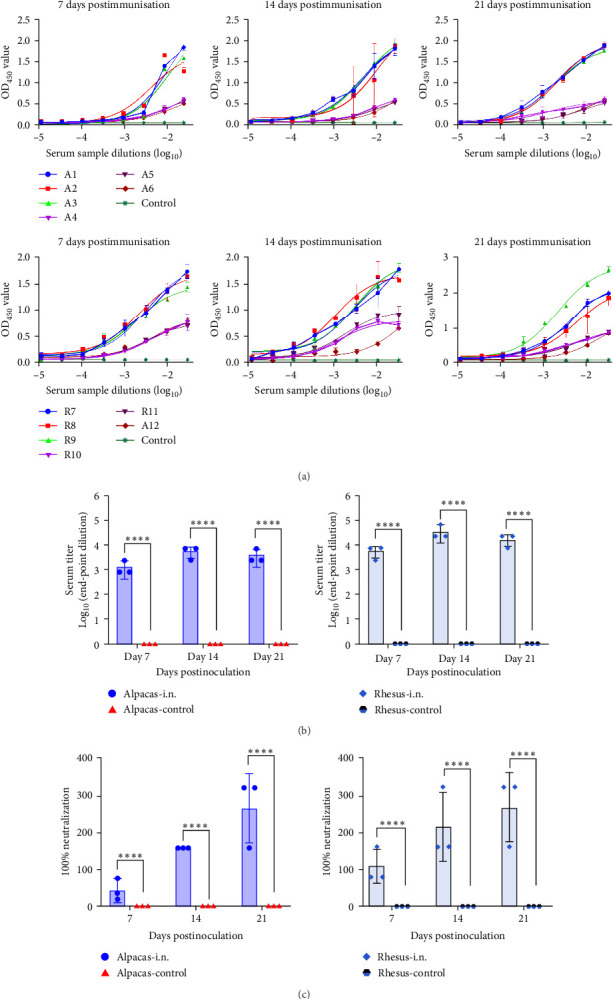
Humoral immune response triggered by rVSVΔG-MERS-S in alpaca and rhesus monkeys. (a) OD_450_ values detected when the serum was diluted in a tenfold ratio via ELISA. (a) Alpaca and Rhesus monkeys immunization schedule. A single dose of rVSVΔG-MERS-S was given via the IN spray route (10^6^ TCID_50_/mL, 100 μL, *n* = 3 per group). Blood was collected at 7, 14, and 21 dpi, and humoral immune responses were measured. The same volume of DMEM was used for the control group. (b) Binding antibodies on days 7, 14, and 21 postvaccination. (c) Neutralizing antibodies on days 7, 14, and 21 postvaccination. Significance levels were defined as *⁣*^*∗∗∗∗*^*p* < 0.0001.

To confirm the hypothesis that functional DPP4 receptors play a role in the efficacy of mucosal vaccines, the immunogenicity of rVSVΔG-MERS-S was evaluated in MERS-CoV-susceptible animals, rhesus monkeys, and alpaca. These animals were given a single nasal spray dose of rVSVΔG-MERS-S (10^6^ TCID_50_/mL, 1 mL per animal). In these animals, rVSVΔG-MERS-S was immunogenic and induced VNAs and antigen-specific antibodies (Figure 6a–c). In these animals, binding antibody and VNA mounted at 2 and 3 weeks postvaccination, respectively, which were significantly higher than the control group (*p* < 0.0001) (Figure 6b,c). These results suggest that rVSVΔG-MERS-S is immunogenic in MERS-CoV-susceptible animals.

### 3.7. Potential Amino Acid Sites in DPP4 Among Species That Affect the Effectiveness of Mucosal Immune Vaccines

**Figure 7:**
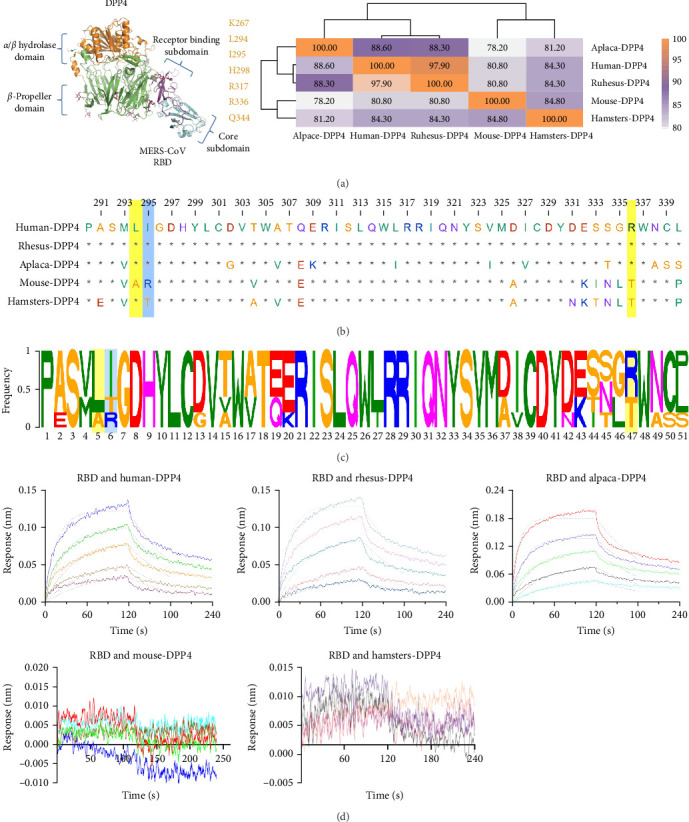
Alignment of amino acid sequences of hDPP4 from mice, rhesus monkeys, and humans. (a) Structural analysis of key binding sites between hDPP4 and the MERS-CoV RBD [14]. (b) Homology analysis of DPP4 receptors in mice, hamsters, alpaca, rhesus monkeys, and humans. (c) Sequence alignment and differential analysis of DPP4 receptors in mice, rhesus monkeys, and humans. (d) Affinity binding curves of DPP4 with the MERS-CoV RBD via BLI.

Owing to the structure of the MERS-CoV spike RBD in complex with the human receptor DPP4 (Figure 7a) [14], there are seven binding sites, including K267, R336, L294, I295, H298, R317, Q344, and R317. Here, alignment of DPP4 receptor data from humans, mice, rhesus monkeys, and humans was performed. Compared with DPP4 receptors from humans, those from rhesus monkeys presented a homology of more than 97%, followed by those from alpaca, with a homology of 88%. In contrast, mice and hamsters presented relatively low homology, at ~80% (Figure 7b). According to the alignment, three key amino acid residues may be related to this difference in sensitivity to mucosal vaccines: L294, I295, and R336. (Figure 7c). The results of the binding affinity assay measured by BLI (Figure 7d) revealed that the *KD* values between the RBD and human/rhesus monkey/alpaca dpp4 were 55.1 nM, 47.8 nM, and 58.4 nM, respectively. No binding affinity was observed between the RBD and mouse/hamsters-DPP4.

## 4. Discussions

Mucosal vaccines have been popular over the past few decades because they offer several competitive advantages. In addition to systemic immune responses, these needle-free mucosal vaccines trigger effective local immune responses, such as SIgA and tissue-resident memory (TRM) cells, coupled with the innate immune response (trained immunity), which renders a line of defense at the mucosal site [15, 16]. Compared with systemic immune responses, the protection conferred by these mucosal immune parameters may be even broader [17, 18]. Additionally, mucosal vaccines can elicit immune responses at a minimal dose of antigens, which reduces the occurrence of first-pass metabolism and the risk of anaphylactic shock. Moreover, mucosal vaccines are safe and convenient, facilitating massive vaccination in epidemic/pandemic settings for both humans and animals [19–21]. Given these merits, mucosal vaccines are undoubtedly the primary choice for treating emerging infectious diseases.

However, several challenges need to be carefully addressed when developing and deploying mucosal vaccines. In particular, species-associated differences include host susceptibility, microbiota, nutritional conditions, regional differences, and issues that are far more undiscovered [22–24]. Mucosal tissues represent entry points for pathogen, infection, and microorganism–antigen interactions. Specifically, antigens encounter an endogenous microbiota-conditioned and regulated mucosal immune system, which affects immunity and the response to infection [25, 26]. For example, owing to differences in the function and distribution of the angiotensin converting enzyme 2 (ACE2) receptor, the efficacy of viral vector-based mucosal coronavirus disease 2019 (COVID-19) vaccines tested in WT mice is opposite that of NHPs or humans [4, 7–10]. Conflicting results in animal studies may even influence the further steps of vaccine development. For example, Merck initiated two phase I human clinical trials of viral vector-based COVID-19 vaccines, including the VSV and measles virus vectors [9, 10, 27]. Both reported limited immunogenicity in humans, which could be largely attributed to the suboptimal delivery route selected from animal experiments. These results underscore the importance of selecting appropriate animal models under given conditions.

Beyond quantifying systemic immune parameters including binding antibody titers and neutralizing antibody potency, this investigation revealed hDPP4-dependent secretory SIgA induction, a hallmark of functional mucosal immunity. SIgA, the predominant immunoglobulin at mucosal interfaces, demonstrates multifaceted protective roles through pathogen neutralization, epithelial adherence blockade, and microbiota modulation [28–31]. Notably, longitudinal human studies have established its superior durability and cross-protective capacity against antigenically divergent pathogens compared to systemic antibodies, positioning it as a critical correlate of mucosal vaccine efficacy. Mechanistically, our findings align with clinical benchmarks through synergistic axes, hierarchical activation of antibody-secreting cell lineages in mucosal compartments, and balanced Th1/Th2 polarization evidenced by coordinated IFN-γ and IL-4 secretion.

These multilayered parallels between hDPP4-transgenic models and human mucosal immunity suggest conserved regulatory mechanisms underlying vaccine-induced protection [32]. However, critical knowledge gaps persist regarding tissue-resident immunity: the ontogeny and maintenance of mucosal-resident memory T cells (Trm) following vaccination, spatial coordination between innate lymphoid cells (ILCs) and dendritic cell subsets in antigen sampling, and epigenetic reprogramming of local pCs for prolonged SIgA production. Future studies employing single-cell multiomics and spatial transcriptomics should elucidate these mechanisms while validating the model's translational fidelity through comparative analysis of human mucosal challenge data.

To reveal the underlying mechanism of the sensitivity differences in animal models, a VSV-based mucosal vaccine for MERS-CoV was prepared. Rescue and operation are performed via reverse genetics approaches [33, 34]. Surface envelope glycoproteins are responsible for host cell binding and invasion. VSV delta G strategies were designed for foreign gene delivery. Specifically, the glycoprotein gene of the viral vector was deleted and replaced with MERS-S [35]. In this strategy, the targeted glycoprotein can be displayed on the surface of the recombinant virus. Accordingly, the cell and tissue tropism of the recombinant virus is largely dependent on foreign glycoproteins. This design strategy renders the recombinant virus ideal for biological growth and minimization of antivector immunity for a mucosal vaccine candidate [36–39]. More importantly, rVSVΔG-MERS-S could simulate the natural infection process of MERS-CoV. These characteristics support the use of rVSVΔG-MERS-S as a mucosal vaccine candidate for further evaluation.

In commonly used experimental animal models, C57BL-6J mice and Syrian hamsters, rVSVΔG-MERS-S was insensitive after nasal spray inoculation. This may be due to the low sensitivity of these species to MERS-CoV infection, which hampers the entry and host recognition of the antigens. The results were subsequently evaluated in hDPP4 transgene-expressing mice and hDPP4-transduced hamsters [34]. In contrast, rVSVΔG-MERS-S was highly immunogenic in hDPP4 mice and hDPP4-transduced hamsters, which corresponded to effective APC activation and antibody production-associated cell proliferation, enrichment of antiviral pathway genes, and robust cellular and humoral immune responses. As expected, in naturally susceptible animals of MERS-CoV, alpaca, and rhesus monkeys, rVSVΔG-MERS-S was also immunogenic in a similar manner [40, 41].

To better understand the molecular basis by which DPP4 contributes to sensitivity differences, DPP4 sequences in mice, hamsters, alpacas, rhesus monkeys, and humans were aligned. The field of view focuses on the receptor binding motif, which involves the specific interaction (mediated mainly by hydrophilic residues) of the RBD of the MERS-CoV spike protein and the DPP4 receptor [42]. In Beta-CoVs, the external receptor binding motif region can vary. Three key amino acid mutations in the receptor binding motif, namely, 294A, 295R, and 336T, may determine the insusceptibility and responsiveness of mice to mucosal vaccines. In accordance with the efficacy of the vaccine, the binding affinity assay revealed that the RBD efficiently binds to human/rhesus monkey/alpaca DPP4 but poorly binds to mouse/hamsters-DPP4. These results are highly important for the application of novel technologies to establish sensitive models, such as CRISPR-Cas gene editing, and subsequent application in the establishment of mucosal immune vaccine evaluation models [41].

Overall, this proof-of-concept study provides evidence that functional receptors in animal models are a prerequisite for effective infection and can be used to evaluate mucosal vaccines. These results could guide the development and selection of appropriate animal models for the evaluation of mucosal vaccines and, as a result, accelerate breakthroughs in the field of mucosal vaccines.

## Data Availability

The data that support the findings of this study are available from the corresponding author upon reasonable request.

## Supporting Information

Supporting Information Figure S1. Safety evaluation of rVSVΔG-MERS-S. Table S1. Animal grouping and experimental design. Table S2. Antibodies used for flow cytometry.
