# Supplementary material for: The Functional DPP4 Receptor Is an Indispensable Factor Mediating the Immune Performance of Mucosal Vaccines for Middle East Respiratory Syndrome
Source: Transbound Emerg Dis. 2025 Jun 10;2025:2303502. doi: 10.1155/tbed/2303502 (PMC12173559; doi:10.1155/tbed/2303502)
Supplement: Supporting Information — Figure S1. Safety evaluation of rVSVΔG-MERS-S. Table S1. Animal grouping and experimental design. Table S2. Antibodies used for flow cytometry. [file 2303502.f1.docx]

Supplemental materials

**The functional DPP4 receptor is an indispensable factor mediating the immune performance of mucosal vaccines for Middle East respiratory syndrome**


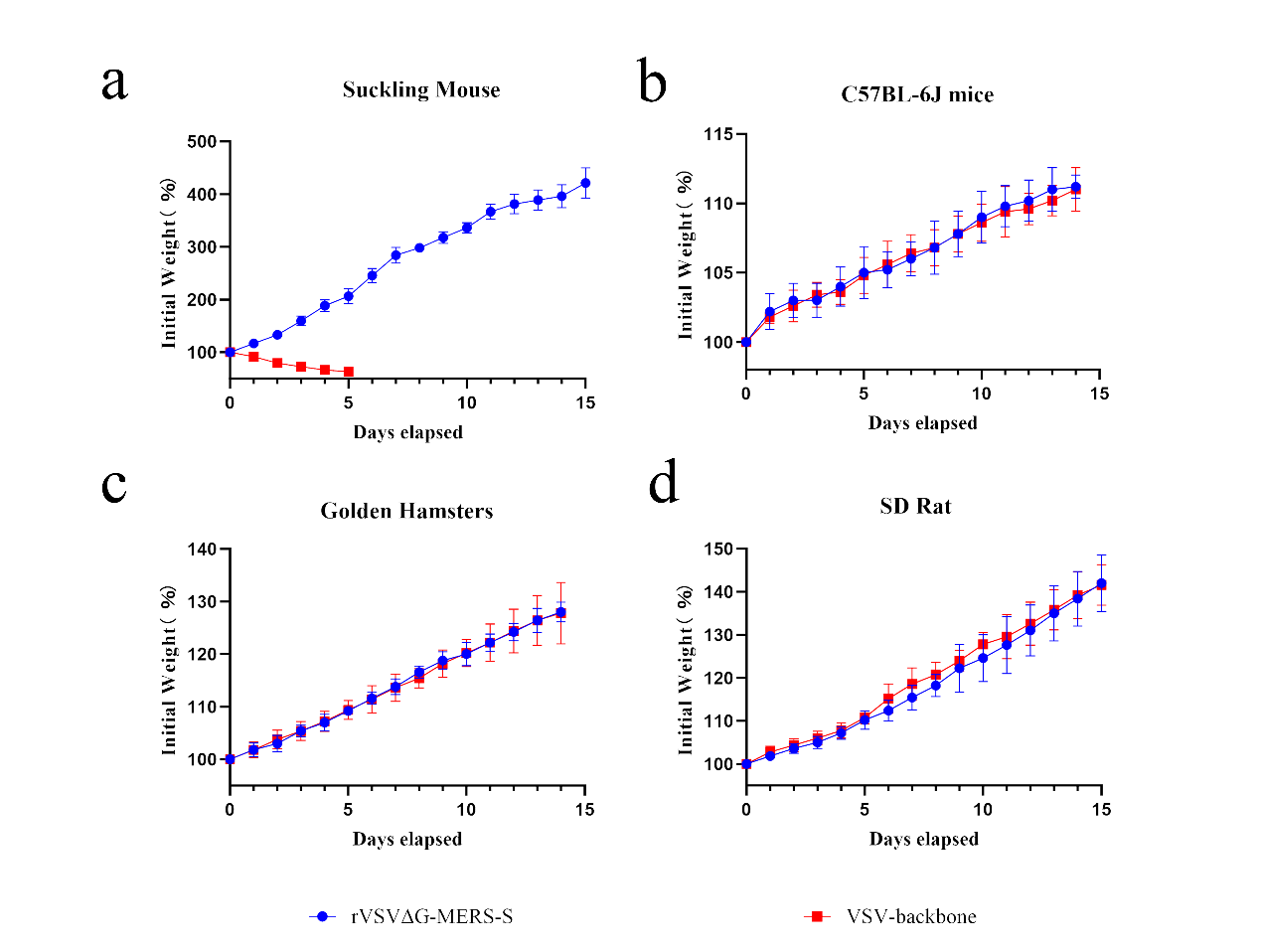
Zhenshan Wang^1,2#^, Xiaojun Hu^1,2#^, Shen Wang^2#^, Hongyu Sun^1,2^, Yongkun Zhao^2^, Na Feng^2^, Tiecheng Wang^2^, Guixue Hu^1^, Jianzhong Wang^1*^, Xianzhu Xia^1,2,3*^, Feihu Yan^2*^

**Fig.S1** Safety evaluation of rVSVΔG-MERS-S. **a-d** Fourteen days successive observation of weights in sucking mice, C57BL-6J mice, Golden hamsters, and SD rat. (Related to Fig. 1)

**Table S1 Animal grouping and experimental design.**

| Animals | Quantities | Treatment | Sample collected | Experimental purpose |
| --- | --- | --- | --- | --- |
| Suckling Mice | n=5 | VSV-Backbone IP | / | Safety Assessment |
| Suckling Mice | n=5 | rVSVΔG-MERS-S IP |  |  |
| C57BL-6J mice | n=5 | VSV-Backbone IP |  |  |
| C57BL-6J mice | n=5 | rVSVΔG-MERS-S IP |  |  |
| Hamsters | n=5 | VSV-Backbone IP |  |  |
| Hamsters | n=5 | rVSVΔG-MERS-S IP |  |  |
| SD Rat | n=5 | VSV-Backbone IP |  |  |
| SD Rat | n=5 | rVSVΔG-MERS-S IP |  |  |
| hDPP4 mice | n=15 | rVSVΔG-MERS-S IN | Bronchoalveolar lavage fluid (n=5 at each time point) | SIgA and cytokines detection |
|  | n=15 | DMEM IN |  |  |
| C57BL-6J mice | n=15 | rVSVΔG-MERS-S IN |  |  |
|  | n=15 | DMEM IN |  |  |
| hDPP4 mice | n=5 | rVSVΔG-MERS-S IN | Serum | Antibody detection |
|  | n=5 | DMEM IN |  |  |
| C57BL-6J mice | n=5 | rVSVΔG-MERS-S IN |  |  |
|  | n=5 | DMEM IN |  |  |
| hDPP4-hamsters | n=5 | rVSVΔG-MERS-S IN |  |  |
|  | n=5 | DMEM IN |  |  |
| hamsters | n=5 | rVSVΔG-MERS-S IN |  |  |
|  | n=5 | DMEM IN |  |  |
| hDPP4 mice | n=5 | rVSVΔG-MERS-S IN | Lung | Transcriptome analysis |
|  | n=5 | DMEM IN |  |  |
| C57BL-6J mice | n=5 | rVSVΔG-MERS-S IN |  |  |
|  | n=5 | DMEM IN |  |  |
| hDPP4 mice | n=10 | rVSVΔG-MERS-S IN | Spleen (n=5 at each time point) | FCM/ELISPOT |
|  | n=10 | DMEM IN |  |  |
| C57BL-6J mice | n=10 | rVSVΔG-MERS-S IN |  |  |
|  | n=10 | DMEM IN |  |  |
| Rhesus monkeys | n=3 | rVSVΔG-MERS-S IN | Serums | Antibody detection |
| Rhesus monkeys | n=3 | DMEM IN |  |  |
| Alpacas | n=3 | rVSVΔG-MERS-S IN |  |  |
| Alpacas | n=3 | DMEM IN |  |  |

IP: intraperitoneal；IN: intranasal

**Table S2 Antibodies used for flow cytometry.**

| **Traget** | **Antibody** | **Catalog** |
| --- | --- | --- |
| **Live/Dead** | **PC5.5-7AAD** | **559925 (BD, NY, USA)** |
| **DC** | **BV421-CD86** | **564198(BD, NY, USA)** |
|  | **PE-CD80** | **561955(BD, NY, USA)** |
|  | **APC-CY7-CD11c** | **561241(BD, NY, USA)** |
|  | **FITC-MHCII** | **562352(BD, NY, USA)** |
| **Tfh** | **FITC-CD4** | **2282605(Thermo Fisher, MA, USA)** |
|  | **APC-CXCR5** | **17-7185-82(Thermo Fisher, MA, USA)** |
|  | **PE-PD-1** | **12-9985-83(Thermo Fisher, MA, USA)** |
| **GcB** | **PE-GL-7** | **2213268(Thermo Fisher, MA, USA)** |
|  | **APC-CD38** | **17-0381-82(Thermo Fisher, MA, USA)** |
| **pCs** | **FITC-CD45R** | **11045286(Thermo Fisher, MA, USA)** |
|  | **PE-CD138** | **46080182(Thermo Fisher, MA, USA)** |
|  | **APC-CD38** | **17-0441-83(Thermo Fisher, MA, USA)** |
| **Macrophages** | **BV605-F40/80** | **743281(BD, NY, USA)** |
|  | **PE-Cy7-CD11b** | **552850(BD, NY, USA)** |
